# Early Clinical and Subclinical Visual Evoked Potential and Humphrey's Visual Field Defects in Cryptococcal Meningitis

**DOI:** 10.1371/journal.pone.0052895

**Published:** 2012-12-21

**Authors:** Anand Moodley, William Rae, Ahmed Bhigjee, Cathy Connolly, Natasha Devparsad, Andrew Michowicz, Thomas Harrison, Angela Loyse

**Affiliations:** 1 Department of Neurology, Greys Hospital, Pietermaritzburg, South Africa; 2 Department of Medical Physics, University of The Free State, Bloemfontein, South Africa; 3 Department of Neurology, University of KwaZulu Natal, Durban, South Africa; 4 Biostatistics Unit, Medical Research Council, Durban, South Africa; 5 Department of Medicine, Edendale Hospital, Pietermaritzburg, South Africa; 6 Cryptococcal Meningitis Group, Research Centre for Infection and Immunity, Division of Clinical Sciences, St. George's University of London, London, United Kingdom; University of Medicine & Dentistry of New Jersey – New Jersey Medical School, United States of America

## Abstract

Cryptococcal induced visual loss is a devastating complication in survivors of cryptococcal meningitis (CM). Early detection is paramount in prevention and treatment. Subclinical optic nerve dysfunction in CM has not hitherto been investigated by electrophysiological means. We undertook a prospective study on 90 HIV sero-positive patients with culture confirmed CM. Seventy-four patients underwent visual evoked potential (VEP) testing and 47 patients underwent Humphrey's visual field (HVF) testing. Decreased best corrected visual acuity (BCVA) was detected in 46.5% of patients. VEP was abnormal in 51/74 (68.9%) right eyes and 50/74 (67.6%) left eyes. VEP P100 latency was the main abnormality with mean latency values of 118.9 (±16.5) ms and 119.8 (±15.7) ms for the right and left eyes respectively, mildly prolonged when compared to our laboratory references of 104 (±10) ms (p<0.001). Subclinical VEP abnormality was detected in 56.5% of normal eyes and constituted mostly latency abnormality. VEP amplitude was also significantly reduced in this cohort but minimally so in the visually unimpaired. HVF was abnormal in 36/47 (76.6%) right eyes and 32/45 (71.1%) left eyes. The predominant field defect was peripheral constriction with an enlarged blind spot suggesting the greater impact by raised intracranial pressure over that of optic neuritis. Whether this was due to papilloedema or a compartment syndrome is open to further investigation. Subclinical HVF abnormalities were minimal and therefore a poor screening test for early optic nerve dysfunction. However, early optic nerve dysfunction can be detected by testing of VEP P100 latency, which may precede the onset of visual loss in CM.

## Introduction

Cryptococcal meningitis (CM) and other opportunistic infections in HIV infected patients continue to be a burden in developing countries despite established antiretroviral drug treatment programmes [1]. A major challenge facing satisfactory management of CM is the late presentation of patients with advanced AIDS at initial presentation. Fifty percent of patients with CM present with neurological complications, of which visual loss is the most disabling in patients that recover [2]. Visual loss is severe, occurs early in infection and is recorded in up to 40% of patients [3]–[6].

Visual loss in CM is well documented, however the pathogenesis remains controversial. Rex et al. have suggested a dual mechanism of early optic neuritis and late papilloedema resulting from optic nerve infiltration and raised intracranial pressure respectively [7]. Nevertheless, conflicting reports of the optic neuritis, papilloedema and the more recent compartment syndrome models abound in the literature [8]–[11]. A definitive model is still lacking but the compartment syndrome occurring along the nerve or at the optic canal level seems most plausible [12], [13]. A better understanding of the pathogenesis of cryptococcal induced visual loss will certainly provide better guidance to management and prevention of blindness in this group. The recommended intervention to prevent visual loss is lowering of CSF pressure, either by serial lumbar punctures, in situ lumbar drain or optic nerve sheath fenestration [8], [14]–[16]. However the likelihood of optic nerve infiltration and the benefit of corticosteroids have not been entirely excluded as treatment option as demonstrated pathologically by Corti et al that fungal infiltration of the optic nerve does occur and by De Schacht et al of the benefit of corticosteroids especially in the setting of immune reconstitution [17], [18].

The Visual Evoked Potential (VEP) is reproducible and the P100 waveform is easily identified and analysed. Full field monocular pattern-reversal VEP is a useful test of pre-chiasmic optic nerve function. VEP findings of prolonged latency and reduced amplitude suggest optic nerve dysfunction, which in the setting of early visual loss in CM points to optic nerve infiltration. Chronic papilloedema may also have similar VEP changes but will not be expected to occur early in the disease. Furthermore, central and centrocecal scotomata early in CM point to optic nerve dysfunction whereas a large blind spot and constricted field will suggest papilloedema related dysfunction. Automated Humphrey's Visual Field (HVF) is not operator dependent and qualitatively offers useful localization of visual pathway dysfunction. Its limitation however is in patients with severe visual loss who cannot be tested. The electrophysiology of optic nerve dysfunction in CM is poorly documented and whether it can contribute towards the discussion of pathogenesis and subclinical disease has not hitherto been explored. Mwanza et al. have demonstrated VEP abnormalities in 57% and 42% of HIV infected patients with and without neurological symptoms, although it is unclear what the burden of CM disease was in these patients [19].

The primary aim of this study was to establish the extent of the electrophysiological disturbance within the optic nerve in patients with CM by examining VEP and comparing with automated HVF. The detection of subclinical disease within the optic nerve by electrophysiological means could potentially identify patients most at risk of developing visual loss. Further correlation with the patients' immune status, visual acuity, optic disc appearance and CSF pressure was made. The secondary aim was to determine if VEP and HVF could contribute to the optic neuritis vs. papilloedema vs. compartment syndrome debate with regard to the pathogenesis of cryptococcal induced visual loss.

## Materials and Methods

Ethical approval was obtained from the University of KwaZulu-Natal and Greys Hospital ethics committees. Informed consent was obtained from 90 patients with CM, confirmed on fungal culture, who were consecutively recruited from February 2008 to January 2011. Patients underwent visual evoked potential testing, visual field testing, neuro-ophthalmological assessment and lumbar punctures, which formed part of their routine work-up for chronic meningitis. Recruitment of patients occurred within 2 weeks of commencement of treatment and usually within 4 weeks of symptom onset of meningitis. CNS co-infection with tuberculosis, toxoplasmosis, syphilis or any other opportunistic infection, when identified, was an exclusion criterion. Lack of co-operation by encephalopathic patients precluded VEP and HVF testing. Flash light emitting diode (LED) goggles VEP was done for 6 patients who could not fixate due to severe visual loss or inattention.

VEP recordings were obtained monocularly. Full field pattern-reversal VEPs were elicited by checkerboard stimuli of large 1 degree (i.e. 60 min of arc) checks and detected using silver electrodes placed over the scalp in accordance with ISCEV guidelines [20]. The P100 latency and peak to peak N80 – P100 amplitude were considered for analysis.

Automated Humphreys Visual Fields were performed using the 30–2 SITA standard protocol. Only pattern deviation fields that fulfilled acceptable reliability indices were included for analysis. Acceptable reliability indices of HVF were fixation losses <33%, false negatives <33% and false positives <33%.

### Stastistics

Visual acuity, VEP latency and amplitude were dichotomized into abnormal and normal groups using standard normal references. A best corrected visual acuity of <6/6 on the Snellen chart, VEP P100 latency of >114 ms and VEP N80-P100 amplitude of <10 µV were considered abnormal. A CSF opening pressure of ≤20 cmH2O was considered normal. One sample t tests were used to compare mean latency and amplitude to laboratory references. Tests for association between groups were analysed using a Chi Square test or Fisher's exact test as appropriate. Statistical analysis was done in STATA, version 12.

## Results

All 90 patients recruited were HIV sero-positive and co-infected with *Cryptococcus neoformans*. All were black African, 50 (55.6%) were males and the mean age of the entire group was 33.5 yrs (range 17–51).

Of the 90 patients recruited, 86 patients had visual acuity testing but 4 were too encephalopathic for testing. Seventy-four patients underwent VEP testing and 47 patients underwent HVF testing. Ten right eyes and 8 left eyes had absent VEP responses therefore 64 right eyes and 66 left eyes were subjected to quantitative VEP analysis for discrete latency and amplitude evaluation. HVF was done on 47 right eyes and 45 left eyes. The disparity was due to an old enucleation of one eye and post traumatic blindness in the other eye (Table 1). Sixteen patients had profound visual loss of <6/60 on whom HVF could not be performed. The results of flash LED-goggles VEP on 6 patients, who were unco-operative, were not reproducible, too unreliable and not included for analysis. Only full field pattern reversal VEPs were included for analysis.

**Table 1:** Frequencies of Abnormal VA, VEP and HVF.

	Visual acuity	VEP	VEP	HVF	HVF
	<6/6	Right Eye	Left Eye	Right Eye	Left Eye
	N (%)	N (%)	N (%)	N (%)	N (%)
Total Number	86 (100)	74 (100)	74 (100)	47 (100)	45 (100)
Normal	46 (53.5)	23 (31.1)	24 (32.4)	11 (23.4)	13 (28.9)
Abnormal	40 (46.5)	51 (68.9)	50 (67.6)	36 (76.6)	32 (71.1)

N – Number, VEP – Visual Evoked Potential, HVF – Humphrey's Visual Field.

While 40/86 (46.5%) patients had decreased best corrected visual acuity (BCVA), profound visual loss of <6/60 was detected in 16/40 (40%) of those patients (19% in total). VEP was abnormal in 51/74 (68.9%) right eyes and 50/74 (67.6%) left eyes (Table 1). Of the 85 eyes with normal visual acuity, 48 (56.5%) had an abnormal VEP parameter (latency and/or amplitude). The mean VEP P100 latency was prolonged and N80-P100 amplitude reduced in both eyes when compared to our laboratory references. The mean VEP P100 latencies for the right and left eyes were 118.9 (±16.5) ms and 119.8 (±15.7) ms respectively, and for the N80-P100 amplitude were 7.4 (±3.9) µV and 7.0 (±3.7) µV respectively. Our laboratory references for these parameters are 104 (±10) ms for VEP P100 latency and 15 (±5) µV for the N80 – P100 amplitude. Mean latency and amplitude differed significantly from hospital references, p<0.001. A further breakdown of these abnormalities show that the P100 latency alone was abnormal in 55/130 (42.3%) eyes, the N80-P100 amplitude alone was reduced in 19/130 (14.6%) eyes and together was abnormal in 9/130 (6.9%) eyes (Table 2).

**Table 2:** VEP latency and amplitude findings in 66 patients.

VEP Latency and Amplitude	VA Right Eye		Total for Right Eye	VA Left Eye		Total for Left eye	VA Combined (%)		Total for both eyes
	Normal	Abnormal	n = 64	Normal	Abnormal	n = 66	Normal n = 85	Abnormal n = 45	n = 130 (100)
Norm lat/Norm amp	18	5	23	19	5	24	37 (78.7)	10 (21.3)	47 (36.2)
Norm lat/Abn amp	9	2	11	7	1	8	16 (84.2)	3 (15.8)	19 (14.6)
Abn lat/Norm amp	12	14	26	16	13	29	28 (50.9)	27 (49.1)	55 (42.3)
Abn lat/Abn amp	1	3	4	3	2	5	4 (44.4)	5 (55.6)	9 (6.9)
Total Abn Latency	13	17	30	19	15	34	32 (50)	32 (50)	64
Total Abn Amplitude	10	5	15	10	3	13	20 (71.4)	8 (28.6)	28

VEP – Visual Evoked Potential, VA – Visual Acuity, Norm – normal, lat – latency, amp – amplitude, Abn – abnormal.

**Figure 1:**
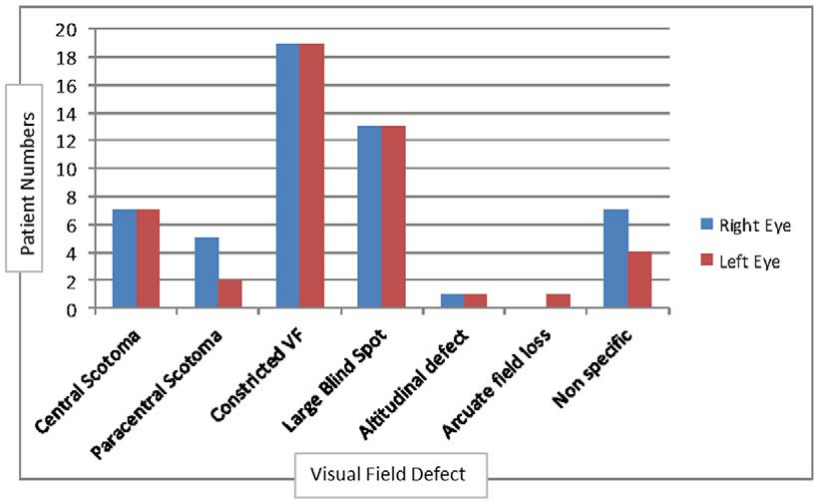
Frequencies of Visual Field Defects. Constricted VF – Constricted Visual Field.

HVF was abnormal in 36/47 (76.6%) right eyes and 32/45 (71.1%) left eyes (Table 1). Peripheral constriction of the visual field and a large blind spot were the predominant defects, followed by central and paracentral scotomata, suggesting the greater impact by raised intracranial pressure over optic nerve infiltration (Figure 1).

Significant correlations were noted between Visual Acuity and VEP latency of the right eye (p = 0.003) and VEP latency of the left eye (p = 0.03,) but not with VEP amplitude (Table 3). When BCVA was abnormal 17/24 (70.8%) and 15/21 (71.4%) of VEP latency was abnormal in the right and left eyes respectively. When BCVA was normal, 13/40 (32.5%) and 19/45 (42.2%) of subclinical VEP latency abnormality was detected. VEP amplitude however did not have a similar relationship, being normal in 19/24 (79.2%) and 18/21 (85.7%) of right and left eyes respectively, when BCVA was abnormal. Subclinical detection of VEP amplitude abnormality was detected in 10/40 (25%) and 10/45 (22.2%) of the right and left eyes respectively.

**Table 3:** Correlation between VEP and VA, CSF Pressure, Swollen optic disc and CD4 count in 66 patients.

		VEP Latency of Right Eye		p value	VEP Latency of Left Eye		p value	VEP Amplitude of Right Eye		P value	VEP amplitude of Left eye		P value
		n = 64			n = 66			n = 64			n = 66		
		Normal	Abnormal		Normal	Abnormal		Normal	Abnormal		Normal	Abnormal	
**Visual Acuity**	Normal	27	13	**0.003**	26	19	**0.03**	30	10	**0.7**	35	10	**0.5**
		(67.5%)	(32.5%)		(57.8%)	(42.2%)		(75%)	(25%)		(77.8%)	(22.2%)	
													
	Abnormal	7	17		6	15		19	5		18	3	
		(29.2%)	(70.8%)		(28.6%)	(71.4%)		(79.2%)	(20.8%)		(85.7%)	(14.3%)	
**CSF Pressure**	Normal	11	9	**0.9**	10	9	**0.6**	17	3	**0.3**	15	4	**0.9**
	Elevated	21	18		19	22		28	11		33	8	
**Swollen** **optic disc**	No	29	18	**0.02**	26	21	**0.047**	36	11	**0.9**	37	10	**0.7**
	Yes	5	12		5	13		13	4		15	3	
**CD4 count**	<50	19	17	**0.6**	16	22	**0.6**	28	8	**0.2**	30	8	**0.9**
	50–100	10	6		9	7		12	4		13	3	
	101–199	1	2		1	1		3	0		2	0	
	>200	1	0		1	0		0	1		1	0	

VEP – Visual Evoked Potential, Visual Acuity refers to Best Corrected Visual Acuity.

Swollen optic disc was correlated with VEP P100 latency for the right and left eyes (p = 0.02, 0.047 resp.) but not with VEP amplitude. No significant correlations were noted between CSF pressure and CD4 count with VEP latency and amplitude (Table 3).

Table 4 demonstrates significant correlation between VEP latency and HVF in both eyes (p = 0.001, 0.0049). BCVA is correlated with HVF of the right eye, but not the left (p = 0.03 vs p = 0.5). Whilst this may reflect the impact of central scotomata on visual acuity testing, figure 1 shows equal frequency of central scotomata in the right and left eyes. Therefore subclinical and asymmetrical optic nerve dysfunction is a possible explanation. When BCVA was abnormal, 17/36 (47.2%) and 10/32 (31.2%) of the right and left HVF were abnormal. Subclinical HVF abnormality was only detected in 1/11 (9.1%) and 2/13 (15.4%) of right and left eyes respectively.

**Table 4:** Correlation between HVF and VA, CSF Pressure, Swollen optic disc, CD4 count and VEP in 47 patients.

		HVF of Right Eye		p value	HVF of Left Eye		P value
		n = 47			n = 45		
		Normal	Abnormal		Normal	Abnormal	
**Visual Acuity**	Normal	10	1	**0.03**	11	2	**0.5**
		(90.9%)	(9.1%)		(84.6%)	(15.4%)	
							
	Abnormal	19	17		22	10	
		(52.8%)	(47.2%)		(68.8%)	(31.2%)	
**CSF Pressure**	Normal	2	9	**0.9**	4	8	**0.4**
	Elevated	8	25		6	24	
**Swollen optic disc**	No	7	4	**0.9**	9	4	**0.8**
	Yes	22	13		20	11	
**CD4 count**	<50	4	19	**0.7**	4	18	**0.14**
	50–100	3	9		5	6	
	101–199	0	2		0	2	
	>200	1	0		1	0	
**VEP latency**	Normal	10	10	**0.001**	9	10	**0.0049**
	Abnormal	1	20		4	19	
**VEP amplitude**	Normal	5	6	**0.1**	3	6	**0.1**
	Abnormal	6	24		10	23	

HVF – Humphrey's visual field, VEP – Visual evoked potential, Visual Acuity refers to Best Corrected Visual Acuity.

## Discussion

Cryptococcal induced visual loss can be devastating and, if neglected, irreversible [21]. Despite the easy availability of antiretroviral therapy in some developing countries, the neuro-opthalmological complications of HIV infection and cryptococcal meningitis are still encountered. Studies have shown that early and intensive management of raised intracranial pressure in CM can reverse the visual loss associated with the disease [8], [9]. Unfortunately, due to the encephalopathic state of most patients with CM and the lack of vigilance by medical personnel in emergency departments visual acuity is not tested or crude testing is done on admission. Early detection of visual impairment is therefore missed and the window of opportunity to reverse optic nerve damage is often lost.

The first aim of this study was to detect the frequency of VEP and HVF abnormalities in patients with CM, correlate these findings with visual acuity, thereby determining the presence of clinical and subclinical disease. The second aim was to determine if this unique VEP and HVF data could contribute to the optic neuritis vs. papilloedema vs. compartment syndrome debate surrounding the pathogenesis of cryptococcal induced visual loss.

Visual impairment was detected in 40/86 (46.5%) of patients with CM, and 16/40 (40%) had profound visual loss of <6/60 (19% in total). VEP abnormalities were detected more frequently, occurring in 68.9% of right eyes and 67.6% of left eyes and subclinical disease in 56.5% (Tables 1 and 2). The predominant abnormal VEP parameter was prolongation of the P100 latency occurring in 42.3% of all eyes (table 2). The mean P100 latency values were 118.9 (±16.5) ms and 119.8 (±15.7) ms for the right and left eyes respectively, mildly prolonged when compared to our laboratory references of 104 (±10) ms but still significant (p<0.001). Prolonged P100 latencies suggest demyelination or conduction block (focal demyelination), as occurs in acute optic neuritis which in the case of CM, a non-demyelinating disorder, will suggest focal pressure effects on the optic nerve. Conceivably the most likely sites of optic nerve compression will be at the optic canal or at sites of dense subarachnoid trabeculae within the optic nerve sheath, providing soft evidence for the compartment syndrome in cryptococcal induced visual loss.

VEP latency strongly correlated with visual acuity and swollen optic disc; when VEP latency was prolonged, visual acuity was reduced and optic disc swelling occurred (Table 3). Such abnormal parameters provide strong clinical and electrophysiological evidence for optic nerve dysfunction in cryptococcal induced visual loss. The fact that 32.5% and 42.2% of right and left eyes respectively with normal acuity had prolonged VEP latency is good evidence for subclinical optic nerve dysfunction. The contribution to prolonged VEP latency made by the HIV virus in advanced HIV infection requires further evaluation. Claims of subclinical VEP abnormalities in 3–49% of HIV infected patients due to retro-chiasmic or occipital cortical neuron loss have not been verified [19], [22]. Mwanza's group did not exclude cryptococcal meningitis and it is likely that the 49% includes patients with cryptococcal meningitis. In Malessa's group of asymptomatic HIV-infected patients, 3% had VEP latency prolongation and 33% had VEP amplitude reduction when CD4 counts were below 100. However in the setting of co-infection with CM, and noting the prominence played by the fungus in visual loss, one has to presume that a large amount of the 56.5% of overall VEP abnormality (latency and amplitude) in our patients was due to cryptococcal co-infection rather than HIV alone (Table 2). A limitation of this study is the failure of comparison to an asymptomatic HIV positive group without CM to determine the impact if any of HIV infection itself. No significant correlations were noted between VEP latency and CSF pressure or CD4 counts (Table 3), possibly due to the relatively small number of patients recruited or that VEP and CSF pressure measurements were not always done at the same time.

VEP amplitude changes occurred less frequently and this was the abnormality in only 14.6% of eyes suggesting that secondary axonal changes were not frequent despite the low mean amplitude of 7.4 (±3.9) µV and 7.0 (±3.7) µV for the right and left eyes respectively.(Table 2). The low frequency of amplitude changes may be a reflection of the somewhat early recruitment of patients from symptom onset (4 weeks). Perhaps repeat testing later in the disease may reveal more secondary axonal change, which is a late phenomenon. Consequently, no significant correlations were noted between VEP amplitude and visual acuity, CSF pressure, optic disc swelling or CD4 counts in early CM. (Table 2).

HVF abnormalities were very frequent in patients who could be tested, occurring in 76.6% of right eyes and 71.1% of left eyes. (Table 1) A major limitation of HVF testing was that patients with profound visual loss (VA <6/60) could not be tested. The predominant field defects were peripheral constriction with large blind spots followed by central and paracentral field defects. (Figure 1) As peripheral constriction with large blind spots is associated with papilloedema-related optic nerve dysfunction, HVF supports raised intracranial pressure being an important cause of optic nerve dysfunction in cryptococcal induced visual loss. The central field defects found suggest intrinsic optic nerve disease or secondary macular dysfunction from severe papilloedema; however an inability to test patients with profound visual loss suggests that the central field defect was probably underestimated in this patient cohort.

HVF is also strongly correlated with VEP latency prolongation (p = 0.001 right eyes and p = 0.0049 for left eyes) (Table 4). Subclinical HVF abnormalities were not as frequent as VEP latency abnormalities and are therefore less sensitive in detecting optic nerve dysfunction in patients with normal visual acuity in CM. No significant correlations were noted between HVF and CSF pressure, optic disc swelling, CD4 counts or VEP amplitude.

The VEP P100 wave is easily recognized on VEP testing and reproducible. Testing of encephalopathic patients was challenging in our cohort of patients with CM. In patients who could be tested, VEP P100 latency was the predominant abnormality and most strongly correlated with decreased visual acuity and optic disc swelling. Furthermore, an appreciable number of patients with normal visual acuity demonstrated P100 latency prolongation suggesting subclinical disease and perhaps a cohort that require close monitoring and aggressive management of raised intracranial pressure to prevent visual loss. The contribution to the P100 latency prolongation made by HIV infection alone needs further investigation. HVF defects were mostly consistent with raised intracranial pressure, even though patients with profound visual loss were unable to be tested.

Prolongation of the P100 latency in early CM lends some support for focal conduction block and hence the compartment syndrome from raised intracranial pressure as a cause for visual loss in these patients. Papilloedema alone which occurs less frequently than visual loss does not account for most cases of visual loss, neither does optic neuritis which is uncommon in the pauci-inflammatory state of CM in HIV infected patients [12]. The neurapraxia caused by the compression at the optic canal or orbital segment of the nerve is potentially reversible by lowering of intracranial pressure rather than immunosuppressant therapy as is used for idiopathic optic neuritis [8], [23].

While this study does demonstrate appreciable P100 latency prolongation even in asymptomatic CM patients, the long term implication of this result can only be answered by longitudinal studies. VEP as a tool to predict visual loss in CM is conceivable and worth further investigation. Furthermore, VEP and HVF provide clinical and subclinical evidence for raised intracranial pressure causing a possible compartment syndrome and optic nerve dysfunction. So can CSF pressure lowering measures in addition to offering an improved overall prognosis prevent blindness in CM by prevention of the optic nerve compartment syndrome rather than merely preventing papilloedema?
